# Formation and stabilization mechanism of mesoscale clusters in solution

**DOI:** 10.1107/S2052252521012987

**Published:** 2022-01-18

**Authors:** Shuyi Zong, Jingkang Wang, Xin Huang, Hao Wu, Qi Liu, Hongxun Hao

**Affiliations:** a School of Chemical Engineering and Technology, Tianjin University, Tianjin 300072, People’s Republic of China; b Collaborative Innovation Center of Chemical Science and Engineering, Tianjin 300072, People’s Republic of China; c School of Chemical Engineering and Technology, Hainan University, Haikou 570208, People’s Republic of China

**Keywords:** crystal nucleation, clusters, stabilization mechanism

## Abstract

To understand the existence of complex meso-sized solute-rich clusters, which challenge the understanding of phases and phase equilibria, the formation and stabilization mechanisms of clusters in solution during nucleation of crystals and the associated physico-chemical rules are studied in detail. An essential part of the mechanism is the formation of long-lived oligomers between solute molecules. By means of density functional theory simulation and nuclear magnetic resonance experiments, this work showed that the oligomers in solution tend to be π–π stacking dimers. Clusters are formed under the combined effect of diffusion and monomer–dimer reaction. The physically meaningful quantities such as the monomer–dimer reaction rate constants and the diffusion coefficients of both species were obtained by reaction-diffusion kinetics and diffusion-ordered spectroscopy results. The evolution of cluster radius as a function of time, and the qualitative spatial distributions of monomer and dimer densities under steady-state were plotted to better understand the formation process and the nature of the clusters.

## Abstract 2

The mechanism for the formation and stability of nucleation precursors of mesoscale clusters is explored and previous work on clusters mainly consisted of phenomenological reports.

## 1. Introduction

Crystal nucleation in solution suggests that crystal nuclei might form inside the solute-rich clusters. The crucial role of the solute-rich clusters as locations of crystal nucleation has been supported by experimental evidence in many systems, such as proteins (Vekilov, 2010 ▸; Kuznetsov *et al.*, 2001 ▸), biominerals (Pouget *et al.*, 2009 ▸; Gebauer *et al.*, 2008 ▸), polymers (Wang *et al.*, 2009 ▸), colloids (Leunissen *et al.*, 2005 ▸; Savage & Dinsmore, 2009 ▸) and small organic molecules (Aber *et al.*, 2005 ▸; Harano *et al.*, 2012 ▸). These clusters are of great interest for a number of reasons. The clusters are essential nucleation sites and the solid aggregation of interest therefore can be obtained by deliberately inducing the formation of clusters (Gliko *et al.*, 2005 ▸; Sleutel & van Driessche, 2004 ▸; Safari *et al.*, 2019 ▸). The formation of clusters may provide a unique way to produce meso-sized particles or gels in industrially relevant quantities as the size distribution of clusters is narrow around the steady-state value. The existence of clusters in solution is spatially and chemically heterogeneous, which is of significance for self-assembly and nano-particle manufacturing (Elbaum-Garfinkle *et al.*, 2015 ▸; Banjade *et al.*, 2015 ▸).

Similar clusters have been studied in our previous work, using the small organic molecule, 2-Cyano-4′-methyl­biphenyl (OTBN) as the model compound (Zong *et al.*, 2020 ▸). There are some phenomenological findings about OTBN clusters in methanol (Zong *et al.*, 2020 ▸): (*a*) only subnanometer molecular assemblies exist in solution when the solute concentration is lower than a certain value, whereas mesoscale clusters appear in solution at higher concentrations; (*b*) the mesoscale clusters have typical size characteristics, that is, they will not grow if they represent the formation of a new, thermodynamically favorable phase; (*c*) clusters appear to be disordered and liquid in nature; (*d*) increasing solute concentration has little effect on cluster size; and (*e*) the solute molecules in clusters move and rotate with lower speed than those in bulk solution. The findings highlight the unusual nature of the clusters. Equilibrium solutions containing meso-sized inclusions seem to be a unique phenomenon and the stabilizing mechanism is not well understood.

The study of clusters in protein systems has greatly promoted the advancement of the cluster stabilization mechanism. Vekilov and coworkers proposed a possible mechanism for the formation of stable protein clusters whereby the clusters might not consist of natural proteins, but some complexes, such as sub-populations of oligomers or mis-folded proteins (Pan *et al.*, 2010 ▸; Li *et al.*, 2011 ▸, 2012 ▸). They suggested that, in the initial weak protein solution, the new species is in equilibrium with protein monomers while the condensed state of the new species favors the formation of supercritical clusters, such as oligomer-rich droplets. However, since the density of the secondary substances in the clusters is much higher than the chemical equilibrium concentration of the monomers, the secondary substances have a tendency to transform into monomers in the clusters, thus hindering the growth of the clusters. In this work, application of the oligomer mechanism of cluster formation was attempted in the small organic molecule systems on the basis of our previous research of OTBN clusters (Zong *et al.*, 2020 ▸). Computational methods and nuclear magnetic resonance (NMR) results were used to analyze the molecular self-assemblies of OTBN molecules in solution. The reaction-diffusion kinetics and the relative parameters measured in the experiments were used to study the OTBN solution, and the feasibility of the proposed model was verified.

## 2. Results and discussion

**Figure 1:**
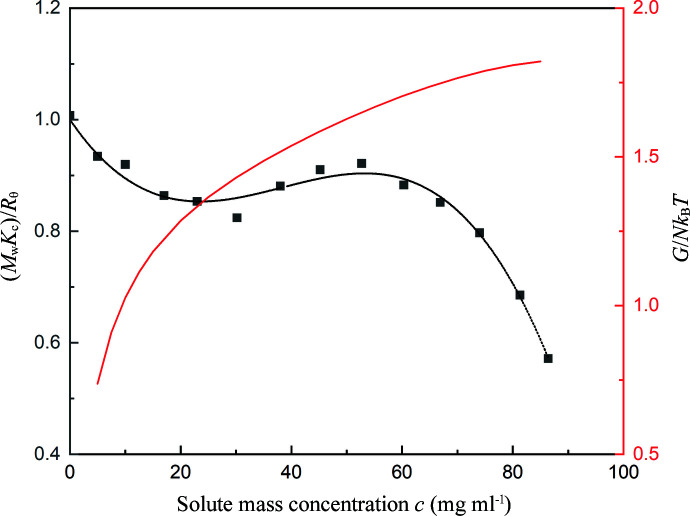
Debye plot of the 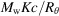 ratio as a function of OTBN mass concentration. Black line: fit of osmotic virial expansion to data. Red line: evaluation of the free-energy density of OTBN–methanol solution according to equation (3).

The thermodynamics of tested OTBN-methanol solutions were characterized by determining the osmotic compressibility 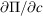 using static light scattering (SLS). The osmotic compressibility can reflect the interactions between the OTBN molecules and be directly related to the ratio *Kc*/*R*_θ_,

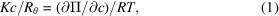

where *R* is the universal gas constant and *T* is the temperature. The *Kc*/*R*_θ_ ratios were measured at various concentrations up to 90.0 mg ml^−1^, which was close to the solubility limit of OTBN in methanol at room temperature. Fig. 1 ▸ displays the mass concentration dependence of the ratio *Kc*/*R*_θ_. The osmotic pressure can be further integrated with the concentration to obtain an expression of free energy

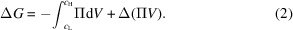

Thus, the free-energy difference between the concentrations *c*_L_ and *c*_H_ for each OTBN molecule is given by (Pan *et al.*, 2010 ▸)

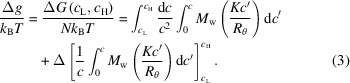

By combining equations (1)–(3 [Disp-formula fd3]) and (16), the parameters 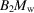 , 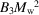 and 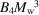 can be obtained and the curve of free-energy excess Δ*g* per OTBN molecule versus concentration *c* can be plotted as Fig. 1 ▸.

As shown in Fig. 1 ▸, increasing solute concentration resulted in an excess of the free energy Δ*g* per solute molecule. Therefore, a large amount of free energy was required to insert a monomer into the clusters, the concentration of which was higher than that of the bulk solution. The single-molecule exchange between a cluster and the bulk solution can be expressed as the sum of two contributions (Pan *et al.*, 2010 ▸): one is the regular Fickian diffusion, and the other contribution comes from the surface tension between the clusters and the bulk solution, which always leads to outward flow of the clusters, even though the clusters are thermodynamically favorable. Thus, the main consideration is the issue of Fickian diffusion. Fick’s law states that diffusion always proceeds in the direction of low concentration. However, in many cases, diffusion proceeds from low to high concentration. This ‘uphill diffusion’ shows that, in essence, the concentration gradient is not always the driving force, and thermodynamics show that the driving force can be the chemical potential gradient. Since the free energy per OTBN molecule increases when the concentration rises, Fickian contribution would push the clusters consisting of monomeric molecules to be consumed. Therefore, for the thermodynamically stable clusters, there must be other species, such as dimers or other higher-order oligomers.

**Figure 2:**
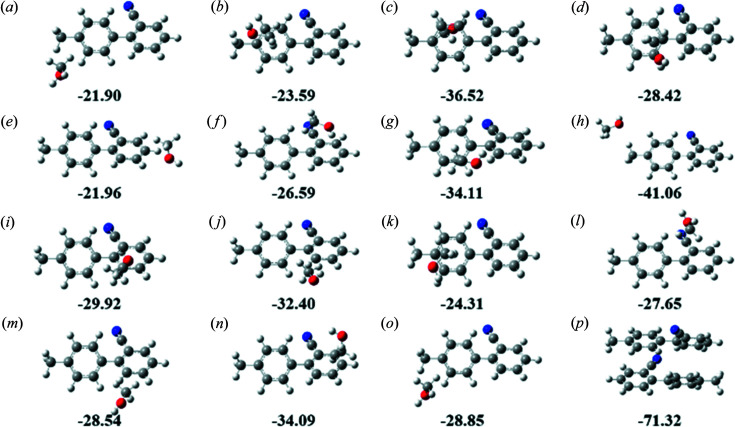
Optimized geometries and binding energies of 1:1 OTBN–methanol complexes and the OTBN dimer for each stable configuration.

**Figure 3:**
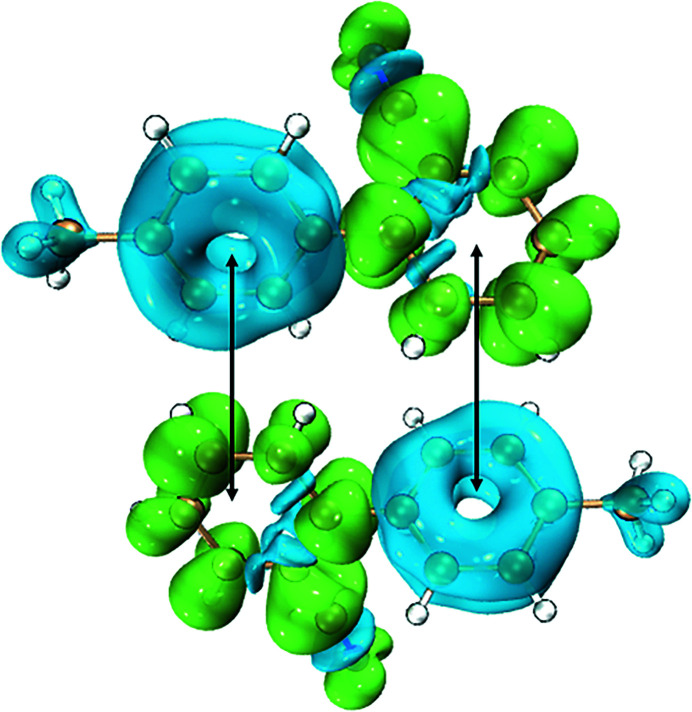
Orbital-weighted dual descriptor isosurface of OTBN dimers.

Dimers are the first aggregates that form during the self-association process, and their structure usually corresponds to the building units of their single crystals (Gaines *et al.*, 2016 ▸; Cruz-Cabeza *et al.*, 2017 ▸). The known crystal structures of OTBN crystallized in methanol are comprised of π–π stacking dimers. In order to investigate whether the π–π stacking dimers are akin to the structure of oligomers in the clusters, computational methods were applied to study the associations of OTBN in methanol. The *Genmer* component in *Molclus* (version 1.9.9.4) was used to generate 20 initial guess structures of 1:1 OTBN–methanol complexes. *Genmer* can put any type or number of monomers (either atoms or molecules) together randomly to generate a specified number of initial cluster configurations. After optimization of the 20 structures using the *Gaussian* package (Frisch *et al.*, 2009 ▸), 5 repetitive structures were removed and the 15 most stable structures were left, as shown in Figs. 2 ▸(*a*)–2(*o*). It can be seen that the main binding sites of OTBN are the benzene ring hydrogens and benzene ring π-electrons. The binding energy of all 1:1OTBN-methanol stable configurations ranges from 21.90 to 41.06 kJ mol^−1^, which are all less than the binding energy of the OTBN dimer, 71.32 kJ mol^−1^ [Fig. 2 ▸(*p*), extracted from the single-crystal structure of OTBN]. Therefore, in methanol solution, the formation of OTBN dimers is thermodynamically favorable. Local electronic structures of OTBN were analyzed in order to understand the intermolecular interactions of the dimers, which result in their relative thermodynamic stability. The isosurface of the orbital-weighted Fukui function of the OTBN molecule was calculated and visualized by *Multiwfn* (Lu & Chen, 2012*b* ▸) and *VMD* (Lu & Chen, 2012*a* ▸) (Fig. 3 ▸). The blue isosurface indicates that the orbital-weighted dual descriptor is negative, which suggests that this region has high nucleophilicity and shows the characteristics of a local Lewis base. The green isosurface indicates that the orbital-weighted dual descriptor is positive, suggesting that this part of the region is vulnerable to nucleophilic attack. As shown in Fig. 3 ▸, large spots of nucleophilic Fukui and electrophilic Fukui functions can be found around the two benzene rings of OTBN. Since the Fukui function is related to the local softness, the matching of Fukui function represents the soft–soft interaction of aromatic stacking (Zhang & Li, 2014 ▸). The interactions control the assembly process of OTBN molecules in solution and can confirm the π–π stacking by the dimers.

**Figure 4:**
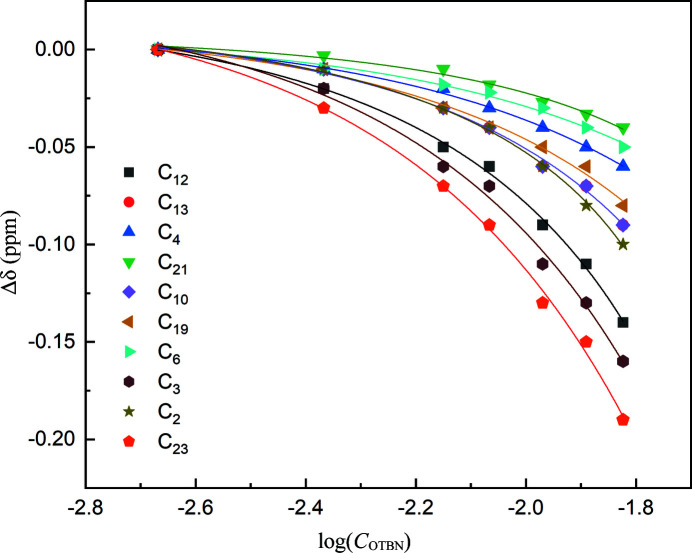
^13^C NMR chemical shift changes of OTBN as a function of concentration in methanol. The lines are the best fit to a dimerization model.

**Figure 5:**
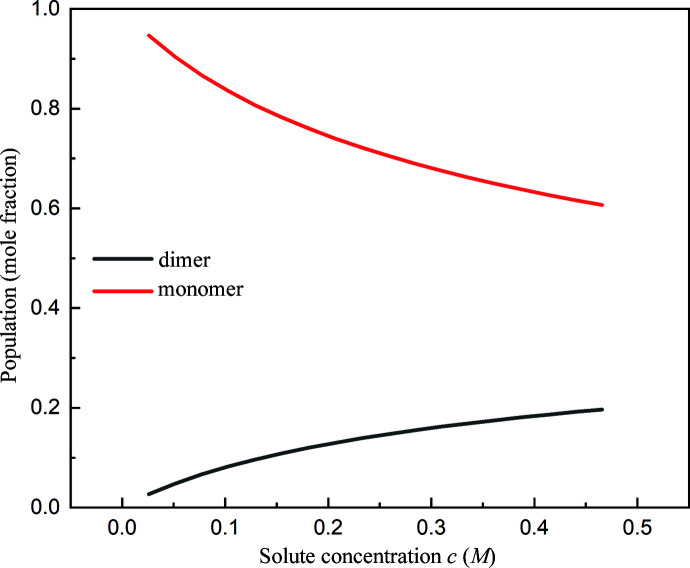
Population of monomers and dimers as a function of total solute concentration.

Intermolecular interactions of OTBN in solution were further investigated by measuring the ^13^C chemical shift. The chemical shift reflects the ensemble average interaction in solution and is highly sensitive to the subtle changes in the local chemical environment of the molecules. As shown in Fig. 4 ▸, almost all ^13^C NMR chemical shifts of OTBN are concentration-dependent in methanol (except for C_18_ whose chemical shifts remain constant over the measured concentration range, not shown in Fig. 4 ▸; the original data are shown in Figs. S1–S2 of the supporting information). ^13^C chemical shifts display a consistent downfield trend as concentration increases, suggesting that the association orientations of OTBN are coincident with the increase of concentration. The deshielding effect of aromatic ^13^C resonance indicates the formation of aromatic stacking associates in solution. The concentration-dependent ^13^C chemical shift change can be well fitted to the dimerization model (see the supporting Information; *R* ^2^ > 0.98; lines in Fig. 4 ▸), yielding dimerization constant *k*_D_ of about 1.147 *M* ^−1^. NMR results further confirmed the dimerization in OTBN solution. The concentration of dimers in solution can then be calculated as follows (Redivo *et al.*, 2019 ▸)

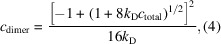

derived by 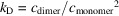 and 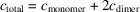 . The calculated populations of monomers and dimers as a function of total solute concentration are shown in Fig. 5 ▸. It can be seen that the dimer population increases with increased solute concentration, reaching the maximum value of about 19.7% at the solubility data. Although OTBN dimers are thermodynamically favorable in solution, the number of dimers in solution is still much lower than that of the monomers. The results of molecular dynamics simulation also confirm that monomers are dominant in solution (Fig. S2).

The above analysis indicates that the OTBN clusters in methanol are composed of mixtures of monomers and dimers. It is consistent with the transient dimer model found in protein systems (Vorontsova *et al.*, 2015 ▸; Byington *et al.*, 2017 ▸), in which dimers or other oligomers are more stable in clusters at higher concentrations than in bulk solutions at lower concentrations, and the clusters are stabilized by the monomer–dimer reaction. This idea can also be applied to the systems of small organic molecules. The 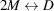 (M refers to monomers, D refers to dimers) conversion was carried out . A population of molecules undergo a chemical reaction to form a new species, which is usually seen as a barrier crossing problem where the molecules with initial free energy *g*_M_ will cross a free-energy barrier at height *g*_B_ and eventually arrive at the new species free energy *g*_D_. The forward reaction rate is proportional to 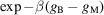 and the reverse reaction rate is proportional to 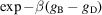 . Hence, the ratio of the forward reaction rate to the reverse reaction rate is proportional to 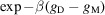 , and a detailed equilibrium can be obtained. This suggests that the dimer-to-monomer reaction should be inhibited in the clusters because clusters are the lower free-energy phase and have higher solute concentration compared with the bulk solution. On the other hand, the monomers in the clusters entail a huge cost of free energy. Therefore, the monomer-to-dimer transition should be strengthened in the clusters. Thus, the cluster should be a dimer-rich phase. When the excess volume free energy of the dimer-rich phase exceeds the upper limit and the clusters become mechanically unstable, the number density of dimers in the cluster was higher than the chemical equilibrium concentration of monomers. There would be a tendency for dimers to convert back to monomers in the cluster, thereby hindering its growth. Taken together, the chemical equilibrium would shift to a new steady state.

The reaction kinetics of monomer density *n*_1_ and the dimer density *n*_2_ can be presented as

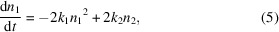




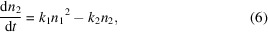

where *k*_1_ and *k*_2_ represent the rate of formation and decay of dimers, respectively. The concentration dependence of *k*_1_ and *k*_2_ is ignored here when concentration variations are small. The constant number density of OTBN molecules is 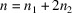 . If monomers are consumed at the rate 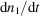 , dimers would be added at the rate 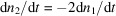 at the same time.

**Figure 6:**
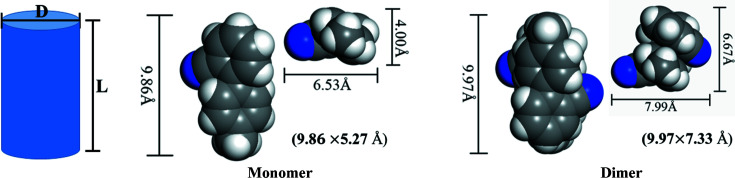
Theoretical dimensions of the monomer and dimer of OTBN. Data in parentheses are the theoretical values fitted to the cylindrical model.

At a sufficient distance from the clusters, there is no net flux of any species and the solution is in local chemical equilibrium, 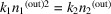 . The reaction-diffusion kinetics are given by a simple generalization of the dynamic density functional theory (DDFT) model (Marconi & Tarazona, 1999 ▸; Archer & Evans,2004 ▸; Lutsko, 2010 ▸), including the formation and decay of OTBN dimers and the transportation of all participating species,

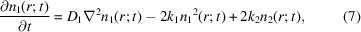




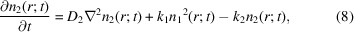

where 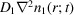 and 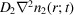 are the contributions to the local monomer/dimer mass balance produced by the exchange of monomers/dimers with adjacent areas; *D*_1_ and *D*_2_ refer to the diffusion coefficients of monomers and dimers, respectively, which can be obtained from diffusion-ordered spectroscopy (DOSY). DOSY can be used to measure diffusion coefficients by fitting the attenuation of NMR spin echo signals intensity caused by the increased strength of the pulse field gradient (Macchioni *et al.*, 2008 ▸). The measured diffusion coefficients can be directly correlated with the size of the diffusant according to the Stokes–Einstein relation (Macchioni *et al.*, 2008 ▸), 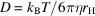 , where *k*_B_ is the Boltzmann constant, *T* is the absolute temperature, η is the solution viscosity, *r*_H_ is the hydro­dynamic radius. Therefore, the diffusion coefficients of dimers and monomers are inversely proportional to their hydro­dynamic radius. The diffusion coefficient of monomeric species was determined to be *D*_1_ = 4 × 10^−11^ m^3^ s^−1^ by measuring the OTBN dilute solution. The hydro­dynamic radius was fit to a cylindrical model (Giuseppone *et al.*, 2008 ▸; Schulze *et al.*, 2014 ▸), leading to the dimensions 3.72 and 4.65 Å for OTBN monomers and dimers, respectively, matching the motifs in Fig. 6 ▸. The diffusion coefficient of dimers was then calculated to be *D*_2_ = 3.2 × 10^−11^ m^3^ s^−1^.

The DDFT equations describe the transportation and mutual conversion of monomers and dimers. At steady state, the left terms of equations (7) and (8) equal to 0, yield

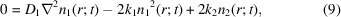




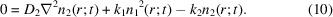

In view of the fact that the clusters of OTBN in methanol are spherical with an average size of about 32 nm (Zong *et al.*, 2020 ▸), the model was solved numerically under the assumption of spherical symmetry. The corresponding general solutions of equations (9) and (10) are given by (Pan *et al.*, 2010 ▸)

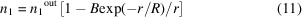




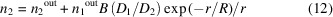

where *B* is an arbitrary constant determined by boundary conditions: 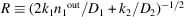 , as the low concentration of dimers in the bulk solution, 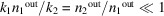 , thus 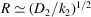 . Then, the decay rate constant of dimers *k*_2_ can be calculated as 125000 s^−1^. Since the cluster radius is not sensitive to the solute concentration (Zong *et al.*, 2020 ▸), the decay rate *k*_2_ should also not depend on the solute concentration, which is consistent with the assumption that the concentration dependence of *k*_2_ was ignored.

Combining the effects of reaction and diffusion, Lutsko & Nicolis (2016 ▸) gave the evolution equation of cluster radius

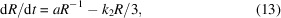




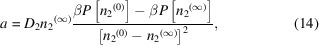

where β = 1/*k*_B_ *T*, *k*_B_ is the Boltzmann constant, *T* is the temperature and *P*(*n*_2_) refers to the pressure for dimers at density *n*_2_. The first term on the right side of the equation represents the driving force for cluster growth, dominated by dimers in solution. The second term can be attributed to the fact that the reaction of the dimer-rich clusters is imbalanced with the monomers; to achieve equilibrium, a proportion of dimers would be converted to monomers, and the monomers will be expelled from the clusters through diffusion, resulting in a decrease in the radius of the clusters. The two contributions are superimposed. Therefore, small clusters tend to grow whereas large clusters tend to shrink until the cluster reaches a stable, fixed size. That is, d*R*/d*t* = 0, leading to stabilization at

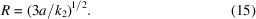

**Figure 7:**
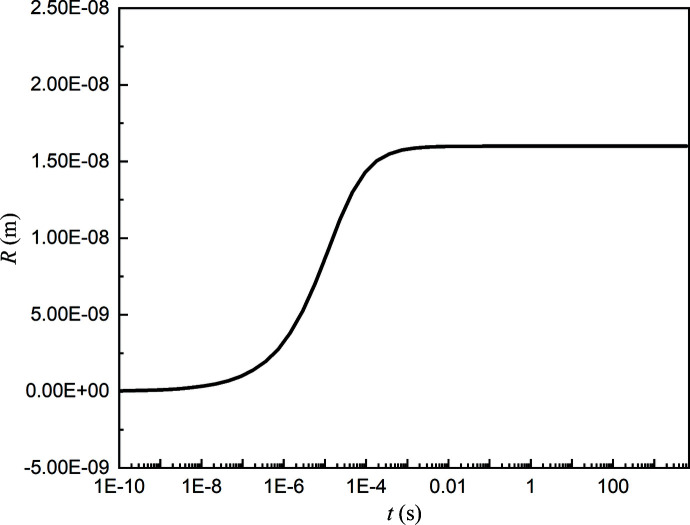
Behavior of the OTBN cluster radius as a function of time.

The parameters can be calculated according to above equations and the evolution of the radius of OTBN clusters can be plotted, as shown in Fig. 7 ▸. We can see that the clusters form in solution in a very short time, growing to a stable size within 10^−3^ s. This is consistent with the experimental observation of clusters forming just after the solute dissolved. The clusters grew gradually at the initial stage, rather than declining from larger clusters to the stable size. This indicates that the clusters are not formed directly by solute dissolution, but are the inevitable result of selection through molecular interactions, especially specific chemical reactions, such as the mutual transformation of monomers and dimers. The force that drives the growth of the cluster is larger than the force that hinders the growth at the initial stage, until the pressure difference between the inside and the outside of the cluster is just enough to balance the volume of the cluster. The behavior of the OTBN cluster radius is also strong empirical evidence for the stability of the OTBN clusters.

**Figure 8:**
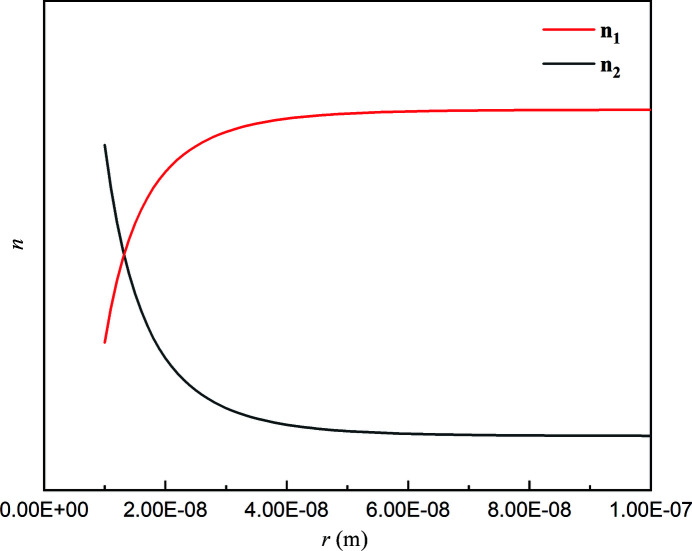
Spatial distribution of monomer density *n*_1_ and dimer density *n*_2_.

In order to further understand the nature of the clusters, the spatial distributions of monomer density *n*_1_ and dimer density *n*_2_ at steady state were plotted. The estimated ratio of 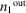 to 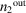 was set between 3 and 35 according to Fig. 5 ▸. We found that the ratio of 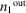 to 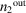 had almost no effect on the trend of *n*_1_ and *n*_2_, and their qualitative curves are shown in Fig. 8 ▸. We can see that the concentration of the monomers in the clusters is very low whereas the concentration of dimers is very high. The high concentration of dimers in the clusters probably contributes to the fact that the clusters are the crucial nucleation sites, as the rearrangement of dimers for nucleation requires a lower energy barrier than the rearrangement of monomers after desolvation. Also note that the concentration gradient of dimers on the cluster boundary is not abruptly reduced, but gradually reduced. That is to say, there was no clear boundary between the clusters and the bulk solution.

## 3. Conclusions

The existence of oligomers in the OTBN clusters in methanol was verified by SLS and free-energy analysis. The contribution of free energy can be regarded as a diffusion driving force that is dependent on the density of monomers and oligomers. The contribution of free energy of a cluster is negative and thus can drive the growth of clusters. The oligomer structure may be similar to the π–π stacking dimers found in single-crystal structures according to DFT simulations. The concentration-dependent NMR shifts combined with dimerization model further confirms the dimerization reaction in OTBN–methanol solution. The dimerization model also provided the population of dimers and monomers and the number of dimers in solution will increase as the concentration increases while the number of monomers is still dominant. The reaction-diffusion kinetics of monomers and dimers can describe the formation and stability of clusters. At the early stage of cluster growth, too many monomers would increase the free-energy cost of the clusters, and the conversion of monomers to dimers would be strengthened by thermodynamic drive. The contribution of free energy can be regarded as a density-dependent diffusion constant, which is negative for the condensed phase, thus driving the growth of clusters. When the dimer content in the cluster exceeds the equilibrium value, a portion of the dimers convert back to monomers in order to balance the clusters and then the monomers move from the center to the boundary and diffuse out of the cluster. This mechanism against cluster growth is triggered by the physical intuition that the reaction should be inhibited when one of the components is in an energetically favorable state. Thus, the clusters were stabilized by the combined effect of diffusion and mutual monomer–dimer chemical reaction. According to the calculated results, the whole formation process can be accomplished in a very short time, within 10^−3^ s. The spatial distribution of monomers and dimers indicates that the clusters have no sharp boundaries. The clusters and the bulk solution can be seen as a spatially open system with the presence of species exchange, which would promote system equilibrium.

## 4. Methods

### 4.1. Static light scattering

Static light scattering (SLS) experiments were performed using the Brookhaven BI-200SM. The concentration range of OTBN solution used in methanol was 10.0–98.2 mg ml^−1^. SLS was determined based on the concentration dependence of the scattered light intensity and the results were plotted as

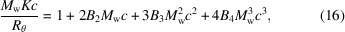

where *R*_θ_ = *I*_θ_/*I*_0_ is the Rayleigh ratio of the scattered to incident intensity and *K* is an optical constant calculated from 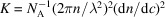 (Berne & Pecora, 2020 ▸), where *N*_A_ is Avogadro’s number, λ is the wavelength, *n* = 1.325 is the refractive index of the solvent and d*n*/d*c* = 0.275 is the derivative of the refractive index *n* with respect to OTBN mass concentration. The necessary determination of d*n*/d*c* was carried out using a Brookhaven Instruments differential refractometer operating at the same wavelength as the laser used for light scattering (Pan *et al.*, 2010 ▸). The measured data were taken from the test angle at 0°.

### 4.2. Computation methods

DFT calculations were carried out using the *Gaussian09* package to investigate interactions in (1:1) molecular complexes of OTBN in methanol (Grimme *et al.*, 2010 ▸). The geometries of OTBN dimers and 1:1 OTBN–methanol complexes were envisaged based on the single-crystal structure of OTBN and optimized by hybrid M06-2x function and 6–31 + G(d,p) basis set with Grimme’s D3 dispersion correction (Boys & Bernardi, 1970 ▸). The Grimme dispersion correction allows a better description of weak interactions, such as van der Waals interactions. The binding energy (Δ*E*_bind_) between two molecules was calculated using the following equation

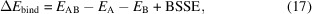

where *E*_AB_ is the energy of the OTBN–methanol complex; *E*_A_ and *E*_B_ are the energies of the isolated monomers OTBN and methanol, respectively; BSSE is the basis set superposition error term calculated to correct the overestimation of binding energies due to the overlapping of basis functions (Bunte & Sun, 2000 ▸).

### 4.3. NMR spectroscopy

All NMR spectra were measured using a 600 MHz liquid NMR spectrometer (Bruker AVANCE III) equipped with a 5 mm QCI Z-gradient cryoprobe. Data were processed and analyzed using *TOPSPIN* software (Bruker). The chemical shifts in the ^13^C spectra were referenced to a fixed methanol-d_4_ peak. DOSY experiments were performed using the bipolar pulse longitudinal eddy current delay pulse sequence at 298 K. The self-diffusion coefficients were measured at a series of concentrations of OTBN in methanol-d_4_, containing 0.3%(*v*/*v*) TMS.

### 4.4. MD simulation

The molecular dynamics were simulated by *Materials Studio* [MS, version 7.0; (Accelrys, 2010 ▸)]. The amorphous cell model composed of OTBN and methanol in terms of molar solubility was chosen in the study, and the cubic periodic cell contained 10 000 molecules. The geometry optimization simulation and molecular dynamics simulation were calculated using the *Forcite* module with the COMPASS (condensed-phase optimized molecular potentials for atomistic simulation studies) force field (Vyalov *et al.*, 2017 ▸) in order to describe the interaction throughout the whole simulation at a fully atomistic level, and the temperature was controlled by a Nosé–Hoover–Langevin (NHL) thermostat. The Smart method combining the conjugate gradient and steepest descent approach was applied to the energy-minimization process, which speeds up the computation. First, the periodic cell was subjected to a 100 000-step *MM*-based geometry optimization to remove the irrelevant contacts. Then, the NVT ensemble dynamic simulation was carried out at the experimental temperature to ensure that the system was in a good state of relaxation and balance. The simulation time was set to 1000 ps and the time step of each dynamic process was set to 1 fs. The van der Waals interaction was computed using an atom-based cutoff distance of 15.5 tÅ and the electrostatic interaction was calculated by the Ewald summation method with an accuracy of 0.418 J mol^−1^. The energy deviation was limited to 209 000 kJ mol^−1^.

## Supplementary Material

Supporting information file. DOI: 10.1107/S2052252521012987/lt5045sup1.pdf
