# Supplementary material for: Formation and stabilization mechanism of mesoscale clusters in solution
Source: IUCrJ. 2022 Jan 18;9(Pt 2):215–22. doi: 10.1107/S2052252521012987 (PMC8895010; doi:10.1107/S2052252521012987)
Supplement: Supplementary file 1 [file m-09-00215-sup1.pdf]

IUCrJ

Volume 9 (2022)

Supporting information for article:

Formation and stabilization mechanism of mesoscale clusters in solution

Shuyi Zong, Jingkang Wang, Xin Huang, Hao Wu, Qi Liu and Hongxun Hao

S1. Dimerization model

The dimerization model is as follows

$$\delta_{obs} = \delta_m \frac{c_m}{c_{tot}} + \delta_d \frac{2k_d c_m{}^2}{c_{tot}} \quad \text{(S1)}$$

$$c_{tot} = c_m + 2k_d c_m{}^2 \quad \text{(S2)}$$

where δ_{obs} refers to the observed chemical shift, δ_m and δ_d refer to the chemical shift of monomers and dimers, respectively, c_{tot} is the total concentration of OTBN, c_m is the concentration of monomer, k_d refers to the dimerization constant.

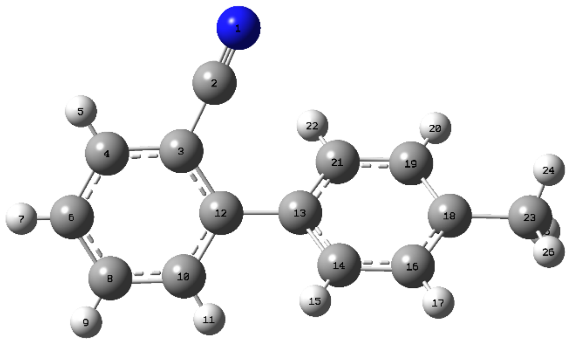

Figure S1 Molecular structure of OTBN.

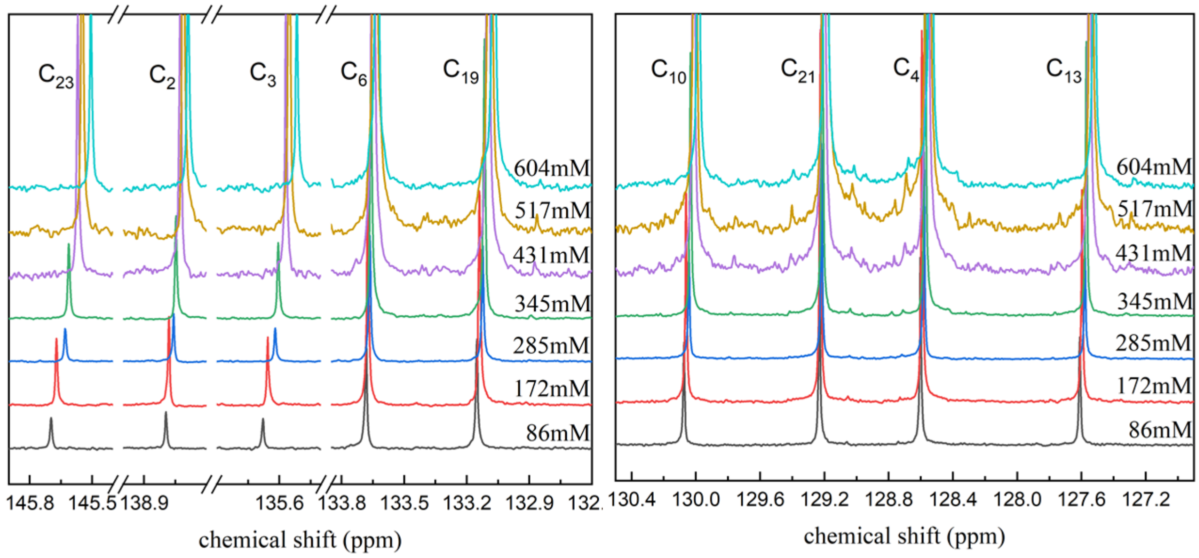

Figure S2 ^{13}C NMR chemical shift changes of OTBN as a function of concentration in methanol.

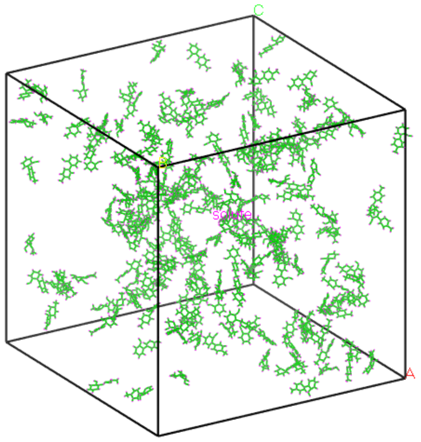

Figure S3 Molecular dynamics results of OTBN in methanol (The solvent molecules were hidden).
